# Fraction and Number of Unemployed Associated with Self-Reported Low Back Pain: A Nation-Wide Cross-Sectional Study in Japan

**DOI:** 10.3390/ijerph182010760

**Published:** 2021-10-13

**Authors:** Kimiko Tomioka, Teruyo Kitahara, Midori Shima, Keigo Saeki

**Affiliations:** 1 Nara Prefectural Health Research Center, Nara Medical University, Kashihara 634-8521, Nara, Japan; mshima@naramed-u.ac.jp (M.S.); saekik@naramed-u.ac.jp (K.S.); 2 Division of Occupational and Environmental Health, Department of Social Medicine, Shiga University of Medical Science, Otsu 520-2192, Shiga, Japan; teruyo@belle.shiga-med.ac.jp

**Keywords:** low back pain, unemployment, gender difference, population attributable fraction, cross-sectional studies

## Abstract

This study examined a cross-sectional association between self-reported low back pain (LBP) and unemployment among working-age people, and estimated the impact of self-reported LBP on unemployment. We used anonymized data from a nationally representative survey (24,854 men and 26,549 women aged 20–64 years). The generalized estimating equations of the multivariable Poisson regression models stratified by gender were used to estimate the adjusted prevalence ratio (PR) and 95% confidence interval (CI) for unemployment. The population attributable fraction (PAF) was calculated using Levin’s method, with the substitution method for 95% CI estimation. The prevalence of self-reported LBP was 9.0% in men and 11.1% in women. The prevalence of unemployment was 9.3% in men and 31.7% in women. After adjusting for age, socio-economic status, lifestyle habits, and comorbidities, the PR (95% CI) for the unemployment of the LBP group was 1.32 (1.19–1.47) in men and 1.01 (0.96–1.07) in women, compared with the respective non-LBP group. The PAF (95% CI) of unemployment associated with self-reported LBP was 2.8% (1.6%, 4.2%) in men. Because the total population of Japanese men aged 20–64 in 2013 was 36,851 thousand, it was estimated that unemployment in 1037 thousand of the Japanese male working population was LBP-related.

## 1. Introduction

The Global Burden of Disease Study 2016 reported that low back pain (LBP) has become the leading cause of years lived with disability [1]. Additionally, LBP has a high economic burden. In Japan, musculoskeletal disorders, including LBP, account for 7.7% of the total medical expenses (about JPY 2.3 trillion) in 2016, ranking third after cardiovascular diseases and neoplasms [2]. According to a recent report in the United States, the annual cost of LBP in 2016 was an estimated USD 134.5 billion of the total healthcare spending [3]. According to a systematic review on the overall costs associated with LBP, indirect costs (sick leave, early retirement, lost household productivity, and presenteeism, etc.) are estimated to be approximately six times higher than direct medical costs [4]. Therefore, LBP is considered to be a major global health problem.

Occupation is a worldwide cause of LBP; globally, 37% of LBP is attributed to occupational risk factors [5]. A large number of studies report that work-related LBP is more prevalent in occupations with heavy lifting, whole-body vibration, forceful movements, and awkward postures [5,6], such as nursing [7], caregiving [8], transport [9], construction [10], and manufacturing [11]. However, it is difficult to determine whether LBP is associated with unemployment through a workplace survey of workers only.

Previous studies reveal that the prevalence of self-reported LBP is significantly higher in the unemployed than in working people [12], and that self-reported LBP is a predictor of health-related job resignations [13] or sickness absences [14]. However, the findings of prior research did not quantify the importance of unemployment associated with LBP as a public health issue. Additionally, previous studies [12,13,14] did not perform stratified analyses by gender. Gender-specific analyses are essential in evaluating the impact of self-reported LBP on unemployment because of the differences in the percentage of employment [14,15] and the prevalence of LBP [5,6] by gender. Therefore, we made an attempt to estimate the gender-specific burden of unemployment attributable to LBP. Our study will be able to demonstrate how important it is to improve the workplace for people with LBP and to enhance LBP care in terms of employment assistance.

In this study, we used anonymized data from the Comprehensive Survey of Living Conditions (CSLC); the CSLC is a representative sample of Japanese people including both the employed and the unemployed, and the CSLC 2013 is the latest data available, as of the end of August 2021 [16]. The aims of this study were twofold. The first was to investigate a cross-sectional association between self-reported LBP and unemployment among working-age people. The second was to estimate the fraction and number of unemployed people associated with self-reported LBP in Japanese working-age people. To clarify gender difference in the association between self-reported LBP and unemployment and its impact, we performed stratified analyses by gender.

## 2. Materials and Methods

### 2.1. Data and Study Participants

**Figure 1:**
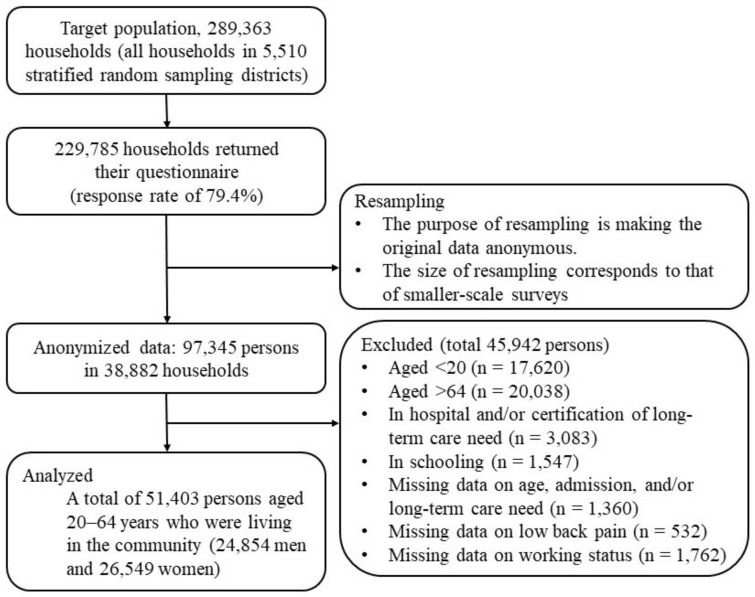
Selection of study participants.

We used anonymized data from the CSLC 2013 conducted by the Ministry of Health, Labour and Welfare of Japan [16]. The CSLC is a cross-sectional nationwide survey which collects data on national lifestyle, health, and welfare. The details of the CSLC 2013 are explained elsewhere [17]. Briefly, the CSLC 2013 targeted all households (approximately 300,000 households) and household members (approximately 740,000 persons) in the 5530 districts stratified and randomly selected from the 2010 census ward. The proportion of respondents was 79.6%. Anonymized data had confidentiality measures such as resampling and top coding. Resampling referred to a survey technique that re-extracts approximately one-sixth of the data from the original CSLC using the same procedure as the original survey. The purpose of resampling was to eliminate concerns about identification of individuals, through shrinking the data and deleting dates of birth and addresses. In the CSLC 2013, questions about lifestyle habits were limited to people aged over 20. Additionally, people in hospital or with a long-term need of care were exempt from answering questions about health status and lifestyles. Therefore, among 97,345 anonymized data, we excluded 45,942 persons from our analyses based on the following criteria: under 20 years old, over 65 years old, in hospital, in school, certification of long-term care need, as well as missing data on age, hospital admission, working status, and/or self-reported LBP. Therefore, this study included 51,403 persons (24,854 men and 26,549 women) (Figure 1). The CSLC was conducted in June 2013, but the employment status was asked in May 2013.

### 2.2. Measurements

#### 2.2.1. Exposure: Self-Reported Low Back Pain (LBP)

The first question was “Did you have any sort of subjective symptoms of a disorder or disease during the past few days?”. Next, persons with subjective symptoms were asked about their symptoms. The second question allowed for multiple answers and presented 42 symptoms. Those who chose “low back pain” in the second question were defined as persons with self-reported LBP. The definition of self-reported LBP in this study was an experience of subjective LBP during the several days prior to being surveyed.

#### 2.2.2. Outcome: Employment Status

Employment status in this study was evaluated using the answer to the question “Did you have any paid work during May of 2013?”. A respondent who answered “Yes” was considered to be employed. On the other hand, a respondent who answered “No” was considered to be unemployed. Only for unemployed persons, did the CSLC ask the question “Are you currently looking for work?”. Using the answers to these questions, unemployed persons were classified into two groups: unemployed looking for work and unemployed not looking for work.

#### 2.2.3. Covariates

Epidemiological evidence suggested that LBP was affected by many factors, reporting that self-reported LBP was common among older age groups [12,18,19,20], widowed or separated individuals [18], people with lower educational level [12,18], people from poorer economic backgrounds [12], drinkers [20], current smokers [6,18,19,20,21], individuals with sleep problems [12,20,22], and those with medical comorbidities [12,18]. Additionally, unemployment was associated with low socio-economic status (SES), cigarette smoking, heavy drinking, and chronic physical illnesses [23]. Therefore, the following variables were included as covariates that could be potential confounders of the associations between LBP and unemployment: age, SES, lifestyle habits, and health status. SES included marital status, family size, housing tenure, equivalent household expenditures, and education. Lifestyle habits included alcohol intake, smoking status, and sleep duration. Health status included comorbidities.

Marital status was categorized into married, never married, and widowed/divorced. Family size was categorized into 1, 2, 3–4, and >4. Housing tenure was dichotomized as owners–occupiers versus renters. Regarding equivalent household expenditures, because there was no gender difference, equivalent household expenditures were divided into three groups by the tertiles as a whole and not by gender: low (lower tertile, JPY 10.5 thousand or less), middle (middle tertile, JPY 10.6–15.6 thousand), and high (upper tertile, JPY 15.7 thousand or more). Regarding education, in Japan, mandatory education was from grade one to nine, and high school education was from grade ten to twelve. Post-high school education was defined as higher education. According to the Japanese school education system [24], education (years of schooling) was categorized into <10 (i.e., junior high graduates), 10–12 (i.e., high school graduates/dropouts), 13–15 (i.e., graduates/dropouts of junior/technical colleges), and >15 (i.e., college graduates and graduate school graduates). For alcohol consumption, in the CSLC, the questionnaire asked about frequency (daily, 5–6 days a week, 3–4 days a week, 1–2 days a week, 1–3 days a month, seldom drink, quit, do not drink). Participants were divided into the following four drinking groups: non-drinkers (quit or do not drink), social drinkers (1–3 days a month or seldom drink), occasional drinkers (1–4 days a week), and almost daily drinkers (more than 5 days a week). Smoking status was categorized into never smokers, ex-smokers, and current smokers. For sleep duration, the CSLC instructed participants to choose from six options for the average amount of sleep per day for the past month: less than 5 h, 5 h or more and less than 6 h, 6 h or more and less than 7 h, 7 h or more and less than 8 h, 8 h or more and less than 9 h, and 9 h or more. A Japanese cohort study reported that more than 7 h of sleep significantly increased the risk of all-cause mortality [25], and in a US population-based study, insufficient sleep duration was defined as less than 6 h per day [26]. In this study, sleep duration (sleeping hours per day) wasd categorized into <6, 6–7, and >7. Comorbidities were defined as participants having at least one disease under treatment for hypertension, diabetes mellitus, cerebrovascular disease, heart disease, and cancer. We confirmed that there were no multicollinearity problems in all covariates (i.e., none of the covariate variables had a variance inflation factor greater than 5.0) and entered all covariates into the final model.

To deal with missing data on the covariates, a category entitled “missing” was used for values that were missing in responses to questions on the covariates [27]. Further details about the covariates are shown in Table S1.

### 2.3. Statistical Analysis

Statistical analyses were performed by using the IBM SPSS Statistics Ver. 24 for Windows (Armonk, New York, NY, USA). The level of significance was 0.05 (two-tailed test). We performed analyses stratified by gender.

Data comparisons between the two groups were tested using the chi-squared test for categorical variables and the *t*-test for continuous variables.

We used the generalized estimating equations of the multivariable Poisson regression model to calculate a prevalence ratio (PR) and a 95% confidence interval (CI) for unemployment. We examined the cross-sectional association between self-reported LBP and unemployment. The independent variable was the presence or absence of self-reported LBP. The dependent variable was unemployment. Additionally, we investigated the relationship of self-reported LBP with unemployment with employment hope. The independent variable was the presence or absence of self-reported LBP, and the dependent variable was unemployment with employment hope.

We used four models to examine the cross-sectional association between self-reported LBP and unemployment. Model 1 was a crude model. Model 2 was adjusted for age. Model 3 was further adjusted for SES including marital status, family size, housing tenure, equivalent household expenditures, and education. The final Model 4 was adjusted as for Model 3 plus lifestyle habits (i.e., alcohol intake, smoking status, and sleep duration) and health status (i.e., comorbidities). For the estimation of PR for those who were unemployed but hoped for employment according to their self-reported LBP status, we used one model; all covariates (i.e., age, SES, lifestyle habits, and health status) were added simultaneously.

To evaluate the potential impact of self-reported LBP on unemployment, we calculated the population attributable fraction (PAF) of unemployment in the Japanese working-age population that could be attributed to the presence of self-reported LBP. We used the Levin formula [28] for the PAF for dichotomous exposures (i.e., the presence or absence of self-reported LBP), and used the substitution method [29] to calculate 95% CI for PAF. The PAF for unemployment was calculated as PAF = [Pe (PR−1)]/[Pe (PR−1) + 1]; where Pe was the prevalence of self-reported LBP in the study participants and PR was the adjusted PR for unemployment of the LBP group controlling for all covariates.

From the population estimation [30], we obtained data on the total population of Japanese men aged 20 to 64 as of June 2013, which was the survey month of the CSLC 2013. Population estimation was the basic statistical data on the country prepared by the Ministry of Internal Affairs and Communications to capture monthly and yearly population data in the middle year of the census. Using the total population of Japanese men aged 20–64 years and the PAF obtained in this study, we estimated how many of the total number of unemployed men aged 20 to 64 would be considered unemployed due to self-reported LBP.

### 2.4. Additional Analyses

Self-reported LBP particular to women included menstrual pain [7] and musculoskeletal pain due to excess strain on the lower back as a result of pregnancy [19]. Therefore, among women, a sensitivity analysis was conducted on the final model, excluding those who answered that they had menstrual pain (*n* = 806) and those who answered that they were going to hospital due to pregnancy (*n* = 154). Moreover, women were exposed to a heavy burden of care responsibilities which included the care of young children and a family member in need of nursing care, and both childcare and long-term care were considered risk factors for LBP [19]. Therefore, among women, a supplementary analysis was performed by adding the presence or absence of preschoolers and the presence or absence of living with persons requiring long-term care to the covariates.

### 2.5. Ethics

Based on Article 36 of the Statistics Act, we received approval of use for academic purposes from the Japanese Ministry of Health, Labour and Welfare (approval number 20009), and were provided data without any information that would identify individuals.

## 3. Results

### 3.1. Characteristics of the Study Participants

A total of 10,730 of the study participants were unemployed, of whom 2323 were men and 8407 women. The prevalence of unemployed women (31.7%) was more than three times higher than that of men (9.3%, *p* < 0.001). The prevalence of self-reported LBP during the past few days was 9.0% in men and 11.1% in women, showing a significant gender difference (*p* < 0.001).

Among both genders, compared to individuals in employment, unemployed persons were significantly older, more likely to spend less, to have a low education, to have comorbidities, and to sleep longer, while they were less likely to be daily drinkers and current smokers (Table 1).

### 3.2. Association between Self-Reported LBP and Unemployment by Gender

Among men, in the crude model (Model 1), people with self-reported LBP tended to have a higher prevalence of unemployment, compared to those without self-reported LBP (crude PR = 1.45, 95% CI 1.29 to 1.63). After the adjustment for age (Model 2), this association was attenuated but remained significant (age-adjusted PR = 1.33, 95% CI 1.19 to 1.49). The additional adjustment for SES brought a consistent result with a significant association (Model 3). In the final model (Model 4), where the data were adjusted for all covariates including lifestyle habits and health status, the association remained significant, showing that men with self-reported LBP had a significantly higher PR for unemployment than men without self-reported LBP (adjusted PR = 1.32, 95% CI 1.19 to 1.47). 

Among women, in the crude model (Model 1), unemployment did not exhibit a significant difference between participants with and without self-reported LBP (crude PR = 1.02, 95% CI 0.96 to 1.08). A non-significant association remained by additional adjustment for age, SES, lifestyle habits, and health status (Model 4), showing that the presence or absence of self-reported LBP was not associated with unemployment in women (adjusted PR = 1.01, 95% CI 0.96 to 1.07) (Table 2).

### 3.3. PAFs for Unemployment Associated with Self-Reported LBP by Gender

The PAF of unemployment due to self-reported LBP was 2.8% (95% CI 1.6% to 4.2%) for men and 0.13% (95% CI −0.48% to 0.77%) for women (Table 2). According to the population estimate released by the Ministry of Internal Affairs and Communications, the total population of Japanese men aged 20–64 as of June 2013 was 36,851 thousand. Therefore, it was estimated that unemployment in 1037 thousand Japanese men aged between 20 and 64 years were related to self-reported LBP.

### 3.4. Relationship of Self-Reported LBP with Unemployed Looking for Work by Gender

After adjustment for all covariates, among women as well as men, people with self-reported LBP had a significantly higher PR among the unemployed hoping for employment than people without self-reported LBP (adjusted PR = 1.49, 95% CI 1.29 to 1.73 in men; adjusted PR = 1.22, 95% CI 1.12 to 1.33 in women) (Table 3).

### 3.5. Additional Analyses for Women

To consider female-specific self-reported LBP, two additional analyses were conducted. First, from a sensitivity analysis limited to women without menstrual pain and pregnancy, similar results were observed. Second, a supplementary analysis was performed with the covariates plus the presence or absence of preschoolers and for living with a care recipient, and similar results were obtained (see Table S2).

## 4. Discussion

Our study had two main findings. First, a significant association between self-reported LBP and unemployment was observed only in men, but not in women, independently of potential confounders such as age, SES, lifestyle habits, and health status; the prevalence of unemployment was significantly higher in men with self-reported LBP than in those without self-reported LBP. Second, we quantified the importance of unemployment associated with self-reported LBP as a public health issue by estimating the PAF and found that self-reported LBP accounted for 2.8% of unemployed men aged 20 through 64 years. In this study, unemployment in 1037 thousand of the Japanese male working population was estimated to be LBP-related. 

Our results are consistent with previous studies, showing that self-reported LBP is associated with unemployment [12,13,31]. This study also reveals the gender differences in this association. However, the mechanisms are unclear. First, previous studies report that women are prone to LBP, but we cannot find reports that women are at an increased risk of leaving their jobs due to LBP. Numerous studies show that women have a higher prevalence of LBP than men [6,11,12,13,32]. For this mechanism, women have a lower level of muscle strength and lung function [33], a lower pain threshold [34], and a higher level of engagement in domestic work than men [19]. In contrast, a significant association between musculoskeletal pain and unemployment was observed only in men [35], and among employees with LBP and/or neck and shoulder pain; being female is significantly associated with a higher risk of work disability but not associated with a risk of unemployment [36]. Next, men who develop LBP may have difficulty finding a new job, because men often work in occupations at high risk of LBP. Hooftman et al. indicated that men are more likely than women to be engaged in back-straining work such as handling heavy objects weighing 20 kg or more and long hours of driving [37]. According to a report on employment services in Japan [38], construction, manufacturing, and transport are among the top five industries with the highest number of mid-career hires. These industries are reported to have a high risk of LBP [9,10,11] and to be male-dominated; the percentage of male workers by industry is 85.6% for construction, 70.5% for manufacturing, and 81.8% for transport [39]. To summarize the above, although women are more vulnerable to LBP than men [19,33,34], unemployed men with LBP may have more difficulty in finding new employment because it is more difficult for men than for women to find work that does not put a strain on the lower back [37,38,39].

Since, as shown in this study, there are a considerable number of men unemployed due to LBP, we should take some measures to address this issue. Our results based on the PAF suggest that if this association is causal, the elimination of self-reported LBP could reduce the risk of unemployment related to self-reported LBP by an estimated 2.8% (95% CI 1.6% to 4.2%) in men. The PAF of 2.8% is equivalent to the estimate that unemployment in 1037 thousand Japanese men aged 20 to 64 years would be LBP-related. Based on the results in Table 3, among both genders, people with self-reported LBP had a significantly higher PR for the unemployed looking for work than people without self-reported LBP. This result suggested that if the LBP problem was resolved, these people may be able to work, regardless of gender. For prevention strategies of LBP, exercise, education, back belts, and ergonomic interventions in the workplace became widespread [40]. With regard to LBP prevention measures in the workplace, it was necessary and effective to take a systematic approach after conducting a risk assessment [41]. Moreover, in Western countries, mass-media campaigns aimed at changing the public’s beliefs about LBP (i.e., changing from the maladaptive belief that rest is important for LBP management because movement creates physical damage to the spine to the sound belief that one should stay active during LBP episodes) were studied with some success [40,42]. Such public health interventions were a possible solution to the LBP problems of unemployed people.

Our study has the following strengths. First, the study’s results are based on a nationally representative sample of Japan, which has a high proportion of respondents: about 80%. This ensures the generalizability of the results of this study. Second, because the CSLC gathers enough information about SES, lifestyle habits, and health status, we are able to control for potential confounding. However, the study does have some limitations. First, because this study is a cross-sectional design, we cannot identify a causal relationship. A longitudinal study based on a nationally representative survey is needed to clarify the causal relationship between self-reported LBP and unemployment. Second, the CSLC asked individuals a question about the presence or absence of subjective symptoms of LBP during the preceding several days. Because many studies evaluate chronic LBP such as the one-year prevalence of LBP [6,10,14,19,22], our study’s results are not comparable with those of previous studies. Furthermore, self-reported LBP may include non-spinal causes of LBP, such as visceral causes (i.e., urinary, gynecological, and digestive disorders), osteoarthritis of the hip, fibromyalgia, obesity, and mood disorders [43]. Because this study has clinical and diagnostic implications, future studies are needed to confirm the association between clinical LBP and unemployment. Third, the definition of having a job (employed) in this study was that the employee had a job with income during May 2013; in the case of a person who did not have a job with income during the same month, his/her status was defined as having no job (unemployed). According to this definition, respondents were classified as unemployed if they worked until April 2013, and as employed if they started working in May 2013. It is unlikely that less than a month of unemployment or work experience would affect self-reported LBP. Although this study aims to investigate the association between self-reported LBP and unemployment, it should be noted that the definition of employment status in this study has a limitation. Fourth, this study uses the data from the 2013 survey, and while this is old data (8 years), it is the latest available data as of August 2021. As the summary tables of the 2016 and 2019 surveys are open to the public [44], we compared the data of the 2013, 2016, and 2019 surveys based on the published spreadsheet in order to verify the representativeness of the results of this study. Because the published tabulation data only lists the number of people who chose LBP as their symptom of most concern, the percentage of those who chose LBP cannot be compared to recent surveys. The percentage of people aged 20–64 with self-reported LBP as the symptom of most concern in 2013, 2016, and 2019 was 4.1%, 4.0%, and 3.8% for men, and 3.6%, 3.4%, and 3.4% for women; the proportion of people with self-reported LBP remains almost unchanged. In contrast, the percentage of unemployed people aged 20 to 64 in 2013, 2016, and 2019 was 11.8%, 10.2%, and 8.8% for men and 33.6%, 30.1%, and 26.4% for women (we should note that the percentages obtained do not match because the criteria for unemployed people to be included or excluded were different between the anonymized data and the aggregated results based on raw data). The proportion of unemployed people declined gradually from 2013 to 2019, particularly for women, where there was a substantial decline. However, it was reported that the number of unemployed people increased for both men and women; there was an especially large increase in the number of females unemployed due to the spread of the COVID-19 since 2020 [45]. Therefore, because the association between unemployment and LBP may be altered by the female workforce participation and a COVID-19-related unemployment, further research is needed to validate the gender-specific association between self-reported LBP and unemployment using the latest data. Fifth, it is difficult to compare the results obtained in this study with the results of previous studies. The reasons for this are that, as far as we know, there is no previous study examining the fraction and number of unemployed people associated with self-reported LBP. Moreover, the definition of self-reported LBP adopted in this study is different from the definition of self-reported LBP in previous studies [6,10,14,19,22]. However, it is consistent with the results of previous studies reporting the association between self-reported LBP and unemployment and suggests that LBP is the most important factor in disability and social costs [1,3,4,12,13,14,31]. Therefore, it is considered that the results of this study are supported by the results of previous studies.

Our findings have important policy implications. In Japan, securing a labor force is an urgent issue due to the rapidly declining birthrate and aging population. The government has implemented measures to encourage women and older people to participate in the labor force and provide employment support for those who could not get a job during the period when it was difficult to find employment after the burst of the bubble economy (currently those in their late 30s to late 40s) [46]. The results of this study show that by taking new measures to solve the LBP problem of unemployed men aged 20 to 64, it is possible to encourage them to return to work and secure the male labor force of a working-age population of about 1 million.

## 5. Conclusions

Independent of relevant confounding factors, a significant association between self-reported LBP and unemployment was observed only in men; men with self-reported LBP were more likely to be unemployed compared to men without self-reported LBP. Additionally, the estimated PAF for unemployment associated with self-reported LBP was about 3.0% in men. Our findings suggested that LBP measures for unemployed men between the age of 20 and 64 could enable some of the LBP-related non-workers to return to the workforce.

## Figures and Tables

**Table 1:** Participant characteristics according to employment status (employed or unemployed), by gender.

	Men (*n* = 24,854)			Women (*n* = 26,549)		
	Employed	Unemployed	*p*-Value *	Employed	Unemployed	*p*-Value *
	(*n* = 22,531)	(*n* = 2323)		(*n* = 18,142)	(*n* = 8407)	
	*n* (%)	*n* (%)		*n* (%)	*n* (%)	
Age: 45 years or older	11,016 (48.9)	1474 (63.5)	<0.001	8635 (47.6)	4690 (55.8)	<0.001
Marital status: not married	7101 (31.5)	1420 (61.1)	<0.001	6975 (38.4)	1526 (18.2)	<0.001
Family size: one (i.e., living alone)	2605 (11.6)	389 (16.7)	<0.001	1558 (8.6)	372 (4.4)	<0.001
Housing tenure: renters	7084 (31.4)	682 (29.4)	0.041	5267 (29.0)	2599 (30.9)	0.002
Household expenditures: low (<10.6)	7045 (31.3)	902 (38.8)	<0.001	5684 (31.3)	2758 (32.8)	0.017
Education: <10 years of schooling	1324 (5.9)	373 (16.1)	<0.001	780 (4.3)	624 (7.4)	<0.001
Alcohol intake: ≥5 days a week	8211 (36.4)	588 (25.3)	<0.001	2524 (13.9)	855 (10.2)	<0.001
Smoking status: current smokers	9077 (40.3)	832 (35.8)	<0.001	2683 (14.8)	920 (10.9)	<0.001
Sleep duration: <6 h a day	8950 (39.7)	627 (27.0)	<0.001	7904 (43.6)	3134 (37.3)	<0.001
Comorbidities: present	2605 (11.6)	471 (20.3)	<0.001	1309 (7.2)	960 (11.4)	<0.001
Self-reported LBP: present	1950 (8.7)	292 (12.6)	<0.001	1996 (11.0)	948 (11.3)	0.515

Note: LBP, low back pain. * *p*-values from chi-squared test. Not married included never married, widowed, and divorced. Household expenditures meant monthly equivalent household expenditures (unit: JPY one-thousand). Comorbidities included hypertension, diabetes mellitus, cerebrovascular disease, heart disease, and cancer.

**Table 2:** Prevalence ratio and population attributable fraction for unemployment associated with self-reported LBP, by gender.

LBP Status		*n*	% of the Unemployed	Model 1	Model 2	Model 3	Model 4	PAF (95% CI)
				PR (95% CI)	PR (95% CI)	PR (95% CI)	PR (95% CI)	
Men (*n* = 24,854)								
	No LBP	22,612	9.0%	1.00	1.00	1.00	1.00	
	LBP	2242	13.0%	1.45 (1.29 to 1.63)	1.33 (1.19 to 1.49)	1.29 (1.16 to 1.44)	1.32 (1.19 to 1.47)	2.8% (1.6% to 4.2%)
Women (*n* = 26,549)								
	No LBP	23,605	31.6%	1.00	1.00	1.00	1.00	
	LBP	2944	32.2%	1.02 (0.96 to 1.08)	0.98 (0.92 to 1.03)	0.98 (0.93 to 1.04)	1.01 (0.96 to 1.07)	0.13% (−0.48% to 0.77%)

Note: CI, confidence interval; LBP, low back pain; PAF, population attributable fraction; PR, prevalence ratio. LBP status means presence or absence of self-reported LBP. Model 1 is a crude model. Model 2 is adjusted for age (per 5-year increase). Model 3 is adjusted for age and socio-economic status (i.e., marital status, family size, housing tenure, equivalent household expenditures, and education). Model 4 is adjusted for the variables in Model 3 plus lifestyle habits (i.e., alcohol intake, smoking status, and sleep duration) and health status (i.e., comorbidities).

**Table 3:** Adjusted prevalence ratios for unemployed looking for work according to presence or absence of self-reported low back pain, by gender.

LBP Status		*n*	% of Unemployed Looking for Work	Adjusted PR * (95% CI)
Men (*n* = 24,854)				
	No LBP	22,612	5.5%	1.00
	LBP	2242	8.0%	1.49 (1.29 to 1.73)
Women (*n* = 26,549)				
	No LBP	23,605	14.3%	1.00
	LBP	2944	16.4%	1.22 (1.12 to 1.33)

Note: CI, confidence interval; LBP, low back pain; PR, prevalence ratio. LBP status means presence or absence of self-reported LBP. * Adjusted for age (per 5-year increase), marital status, family size, housing tenure, equivalent household expenditures, education, alcohol intake, smoking status, sleep duration, and comorbidities.

## Data Availability

Data are available from the Ministry of Health, Labour and Welfare, Japan (https://www.mhlw.go.jp/toukei/itaku/tokumei.html, accessed on 15 September 2021) for researchers who obtain approval to use the anonymous data in accordance with Article 36 of the Statistics Law of Japan.

## Supplementary Materials

The following are available online at https://www.mdpi.com/article/10.3390/ijerph182010760/s1, Table S1: Distribution of covariates by gender, Table S2: Additional analyses among women.
