# Supplementary material for: Fraction and Number of Unemployed Associated with Self-Reported Low Back Pain: A Nation-Wide Cross-Sectional Study in Japan
Source: Int J Environ Res Public Health. 2021 Oct 13;18(20):10760. doi: 10.3390/ijerph182010760 (PMC8536185; doi:10.3390/ijerph182010760)
Supplement: Supplementary file 1 [file ijerph-18-10760-s001.zip › Table S1.pdf]

Table S1. Distribution of covariates by gender

Covariate	Men (n = 24,854)		Women (n = 26,549)		P-value*
	n	(%)	n	(%)	
Age (years)					
Aged 20 to 24	1,249	(5.0)	1,489	(5.6)	0.104
Aged 25 to 29	2,026	(8.2)	2,126	(8.0)	
Aged 30 to 34	2,529	(10.2)	2,642	(10.0)	
Aged 35 to 39	3,209	(12.9)	3,365	(12.7)	
Aged 40 to 44	3,351	(13.5)	3,602	(13.6)	
Aged 45 to 49	3,004	(12.1)	3,104	(11.7)	
Aged 50 to 54	2,846	(11.5)	3,005	(11.3)	
Aged 55 to 59	2,945	(11.8)	3,159	(11.9)	
Aged 60 to 64	3,695	(14.9)	4,057	(15.3)	
Missing	0	(0.0)	0	(0.0)	
Socio-economic status					
Marital status					<0.001
Married	16,333	(65.7)	18,048	(68.0)	
Never-married	7,303	(29.4)	5,779	(21.8)	
Widowed/divorced	1,218	(4.9)	2,722	(10.3)	
Missing	0	(0.0)	0	(0.0)	
Family size					<0.001
1: living alone	2,994	(12.0)	1,930	(7.3)	
Two	5,052	(20.3)	6,311	(23.8)	
3 to 4	13,074	(52.6)	14,214	(53.5)	
≥5	3,734	(15.0)	4,094	(15.4)	
Missing	0	(0.0)	0	(0.0)	
Housing tenure					<0.001
Owner-occupiers	17,088	(68.8)	18,683	(70.4)	
Renters	7,766	(31.2)	7,866	(29.6)	
Missing	0	(0.0)	0	(0.0)	
Monthly equivalent household expenditures (Japanese one-thousand yen)					
Mean ± SD	14.7 ± 7.9		14.6 ± 7.8		0.248
Low: lower tertile	7,947	(32.0)	8,442	(31.8)	0.789
Middle: middle tertile	7,835	(31.5)	8,460	(31.9)	
High: upper tertile	7,958	(32.0)	8,435	(31.8)	
Missing	1,114	(4.5)	1,212	(4.6)	

Table S1. Continued.

Covariate	Men (n = 24,854)		Women (n = 26,549)		*P*-value*
	n	(%)	n	(%)	
Education (years of schooling)					<0.001
Junior high school (≤9)	1,697	(6.8)	1,404	(5.3)	
High school (10–12)	9,419	(37.9)	10,269	(38.7)	
Junior college (13–15)	3,137	(12.6)	7,837	(29.5)	
University or higher (≥16)	8,061	(32.4)	4,271	(16.1)	
Missing	2,540	(10.2)	2,768	(10.4)	
Health behaviors					
Alcohol intake					<0.001
Non-drinkers	5,068	(20.4)	11,039	(41.6)	
Social drinkers	6,009	(24.2)	8,263	(31.1)	
1 to 4 days a week	4,773	(19.2)	3,714	(14.0)	
≥5 days a week	8,799	(35.4)	3,379	(12.7)	
Missing	205	(0.8)	154	(0.6)	
Smoking history					<0.001
Never-smokers	12,705	(51.1)	21,990	(82.8)	
Ex-smokers	1,981	(8.0)	791	(3.0)	
Current smokers	9,909	(39.9)	3,603	(13.6)	
Missing	259	(1.0)	165	(0.6)	
Average daily amounts of sleep					<0.001
7 hours and longer	6,359	(25.6)	6,221	(23.4)	
6 to less than 7 hours	8,797	(35.4)	9,184	(34.6)	
Under 6 hours	9,577	(38.5)	11,038	(41.6)	
Missing	121	(0.5)	106	(0.4)	
Health status					
Comorbidities†					<0.001
Absent	21,702	(87.3)	24,168	(91.0)	
Present	3,076	(12.4)	2,269	(8.5)	
Missing	76	(0.3)	112	(0.4)	

Note: **P*-values from chi-squared test for categorical variables and t-test for continuous variables.

†Persons with at least one disease under treatment for hypertension, diabetes mellitus, cerebrovascular disease, heart disease, and cancer.
