# Supplementary material for: Fraction and Number of Unemployed Associated with Self-Reported Low Back Pain: A Nation-Wide Cross-Sectional Study in Japan
Source: Int J Environ Res Public Health. 2021 Oct 13;18(20):10760. doi: 10.3390/ijerph182010760 (PMC8536185; doi:10.3390/ijerph182010760)
Supplement: Supplementary file 1 [file ijerph-18-10760-s001.zip › Table S2.pdf]

Table S2. Additional analyses among women

LBP status	Sensitivity analysis (n = 25,589)			Supplementary analysis (n = 26,549)		
	n	% of the unemployed	Adjusted PR* (95% CI)	n	% of the unemployed	Adjusted PR† (95% CI)
No LBP	22,996	31.5%	1.00	23,605	31.6%	1.00
LBP	2,593	32.3%	1.00 (0.95-1.06)	2,944	32.2%	0.99 (0.94-1.05)

LBP status means presence or absence of self-reported LBP.

Sensitivity analysis among a sample of women who had neither menstrual pain nor pregnancy.

*Adjusted for age, socio-economic status (i.e., marital status, family size, housing tenure, equivalent household expenditures, and education), lifestyle habits (i.e., alcohol intake, smoking status, and sleep duration) and health status (i.e., comorbidities).

†Adjusted for age, socio-economic status, lifestyle habits, health status, and care of family members (i.e., the presence or absence of preschoolers and the presence or absence of living with persons requiring long-term care).

CI, confidence interval; LBP, low back pain; PR, prevalence ratio.
